# Global research trends and hotspots of fecal microbiota transplantation: A bibliometric and visualization study

**DOI:** 10.3389/fmicb.2022.990800

**Published:** 2022-08-18

**Authors:** Mancai Wang, Xiaofeng Xie, Songbo Zhao, Wei Han, Youcheng Zhang

**Affiliations:** ^1^Department of General Surgery, Lanzhou University Second Hospital, Lanzhou, China; ^2^Medical College, Northwest Minzu University, Lanzhou, China

**Keywords:** fecal microbiota transplantation, research trends, research hotspots, bibliometric analysis, visualization analysis

## Abstract

**Introduction:**

Fecal microbiota transplantation (FMT) has gained considerable attention in a variety of clinical research areas, and an increasing number of articles are being published. It is very critical to reveal the global status, future research trends, and hotspots in the FMT research and application.

**Methods:**

We searched the Web of Science Core Collection up to May 10, 2022, and only articles and review articles about FMT were included finally. CiteSpace 5.8.R3, VOSviewer 1.6.18, Scimago Graphica and Microsoft Office Excel 2019 were used for data analysis and visualization. The results included publication characteristics, Co-authorships analysis, Co-cited analysis, Co-occurrence analysis, and burst analysis.

**Results:**

Eleven thousand nine hundred seventy-two records were used for the analysis and visualization finally, these records were published between 1980 and 2022, and the publication about FMT is increasing year by year. Co-authorship analysis shown that the USA played a key role in this field. After data analysis and visualization, a total of 57 hotspots about FMT were produced. We summarized these hotspots and classified them into 7 grades according to the number of evidence sources. The evidence sources included top 25 of Web of Science categories, top 30 most Co-cited references, top 10 clusters of references, top 25 references with the strongest citation bursts, top 25 keywords with the most occurrence frequency, major 15 clusters of keywords, top 25 keywords with the strongest citation bursts, and top 35 disease keywords.

**Conclusion:**

This bibliometric analysis is expected to provide overall perspective for FMT. FMT has gained increasing attention and interest, there are many hotspots in this field, which may help researchers to explore new directions for future research.

## Introduction

Fecal microbiota transplantation (FMT) is an old and non-conventional therapy comes of age (Kelly, 2013), in which fecal materials from healthy donors are given to patients attempt to cure disease or relieve symptoms (Aroniadis et al., 2019). The concept of FMT is not new in the literature. Some scholars thought that this idea is possibly first proposed in veterinary medicine by the Italian anatomist Fabricius Aquapendente in the 17th century (Borody et al., 2004; Brandt et al., 2012). However, Zhang et al. firmly believes that it is Ge Hong, a well-known traditional Chinese medicine doctor in China, described the use of human fecal suspension by mouth for patients who had food poisoning or severe diarrhea during the Dong-jin dynasty in the 4th century (Zhang et al., 2012). The earliest reports of FMT in the modern literature can be traced back to 1958, in which fecal enema was used as an adjunct in the treatment of pseudomembranous enterocolitis (Eiseman et al., 1958). However, because of the lack of sufficient evidences, FMT has not become a routine therapy in the past few decades (Zhang et al., 2012).

Numerous studies have proved that gut microbiota dysbiosis is closely related to the occurrence and development of various diseases (Aron-Wisnewsky et al., 2021; Chen et al., 2021; Yang et al., 2021). Sufficient evidences shown that FMT is an efficient way of modulating the gut microbiota and introducing a balanced conglomerate of microorganisms (Browne et al., 2021; Du et al., 2021). FMT is already widely practiced as a highly effective treatment for recurrent *Clostridium difficile* infection (CDI; Hui et al., 2019; Hvas et al., 2019; Green et al., 2020; Tixier et al., 2022). A wealth of researches also supported that it may be used to treat other health conditions, including gastrointestinal (Caldeira et al., 2020; Green et al., 2020; Wu et al., 2022), oncological (McQuade et al., 2020; Lythgoe et al., 2022), cardiovascular (Hu et al., 2019; Zhong et al., 2021), autoimmune (Engen et al., 2020; Liang et al., 2021), metabolic (Aron-Wisnewsky et al., 2019; Proença et al., 2020; Hanssen et al., 2021; Manrique et al., 2021), and neuropsychiatric (Evrensel and Ceylan, 2016; Vendrik et al., 2020) diseases, etc. As expected, FMT may herald the puberty of a broad and exciting new branch of human therapeutics (Kelly, 2013).

In recent years, FMT has gained considerable attention in a variety of clinical research areas as described above, and an increasing number of articles are being published. We speculated that there may be many hotspots and focuses in the field of FMT research. However, few attempts have been made to thoroughly assess the scientific output and current status in this topic from a worldwide viewpoint. Therefore, it is very critical to reveal the global status, future research trends, and hotspots in the FMT research and application.

Bibliometric analysis is a statistical method used for the analysis and visualization of key characteristics and research trends in a specific field using online literature databases (Ellegaard and Wallin, 2015; Donthu et al., 2021), it has been widely applied in a variety of fields. Bibliometric analysis is also an effective tool to qualitatively and quantitatively analyze the publications and identify significant research hotspots and trends (Gu et al., 2021a). In this study, we aimed to conduct a comprehensive bibliometric analysis of publications related to FMT, and gain the research hotspots and potential trends, and finally provide useful reference guideline for future researches.

## Materials and methods

### Data search and selection

We systematically searched the electronic database Web of Science Core Collection (WoSCC) up to May 10, 2022. This search was performed using topic term. Search terms included fecal, faecal, feces, faeces, stool, microbiota, microbiome, microflora, bacteria, transplantation, transplant, transfer, enema, infusion, bacteriotherapy. The full search syntaxes were supplied in Supplementary Table 1. Only articles and review articles were included for the analysis and visualization finally.

### Data analysis and visualization

**Figure 1:**
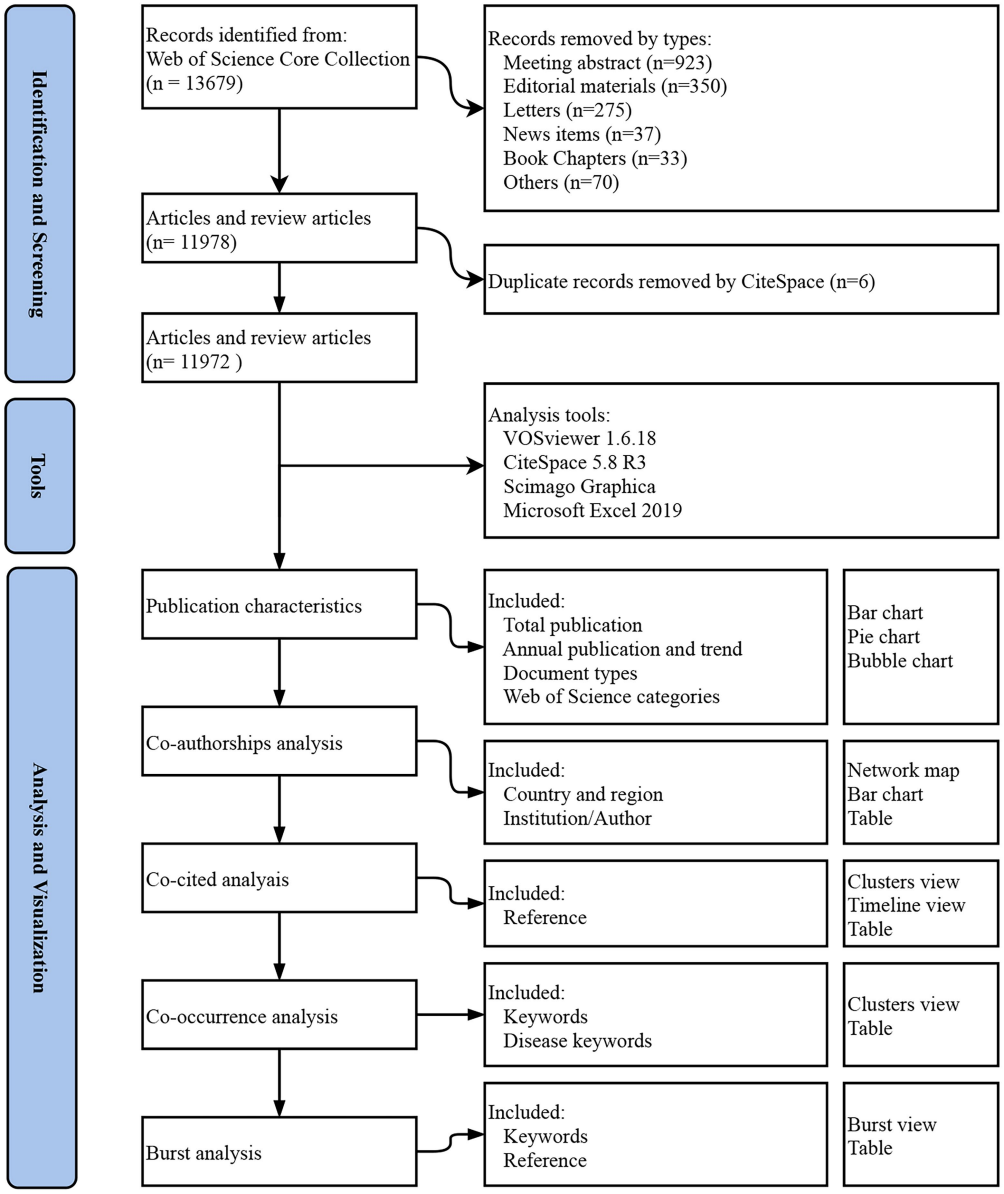
The flowchart of study identification and data analysis/visualization.

We exported the full records and cited references of records from WoSCC. In this study, CiteSpace 5.8.R3, VOSviewer 1.6.18, Scimago Graphica and Microsoft Office Excel 2019 were used for data analysis and visualization. The flowchart of study identification and data analysis/visualization was shown in Figure 1.

Microsoft Office Excel 2019 and Scimago Graphica were used for the analysis and visualization of publication characteristics, which included total publication, annual publication and trend, document types, and Web of Science categories. VOSviewer 1.6.18 and Scimago Graphica were used for Co-authorships analysis and visualization, which included country/region Co-authorships, institution Co-authorships, and author Co-authorships. CiteSpace 5.8.R3 was used for Co-cited analysis, Co-occurrence analysis, and burst analysis. Burst analysis included burst references and keywords analysis. All data in tables was extracted by the VOSviewer 1.6.18.

## Results

### Over characteristics of publication

**Figure 2:**
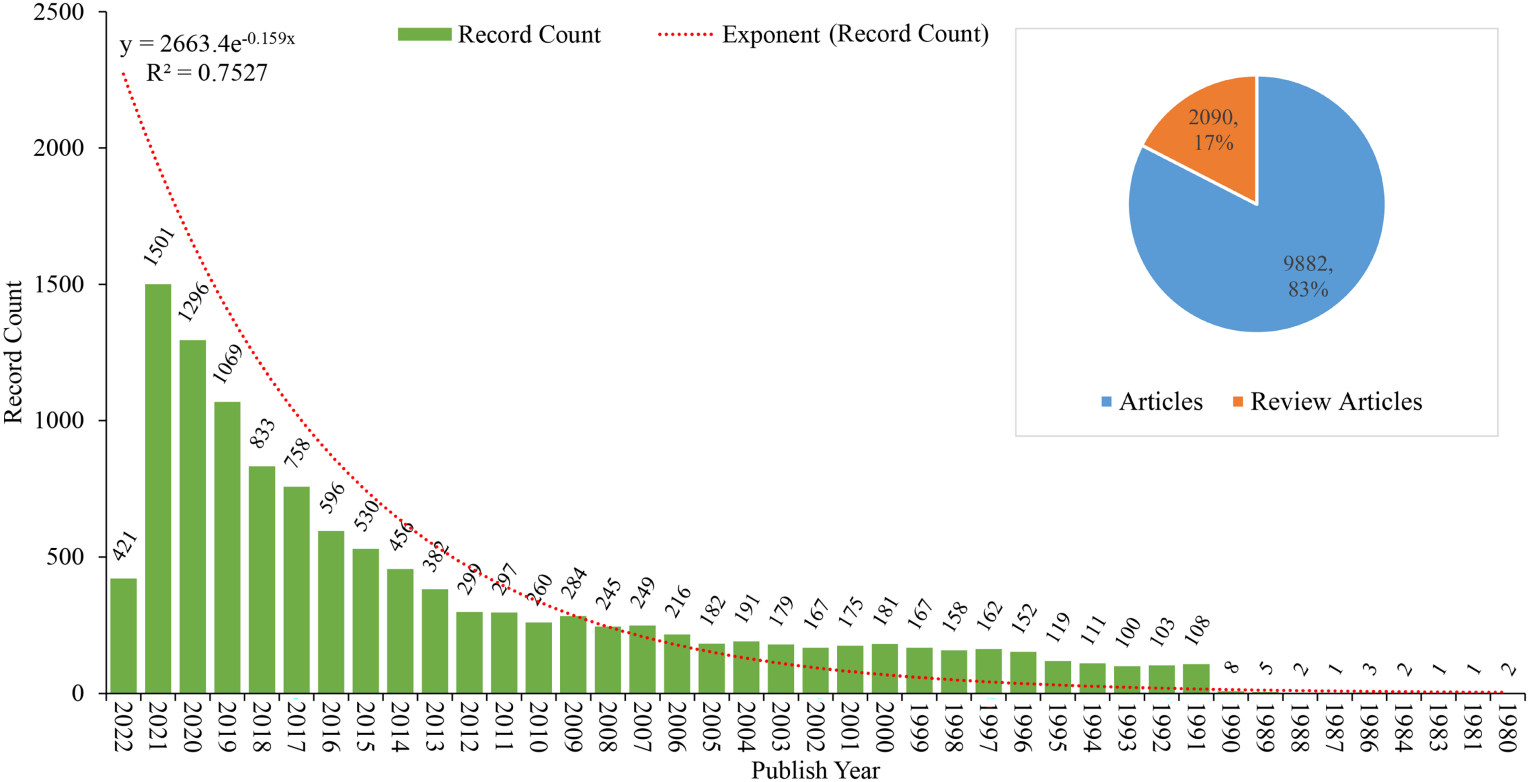
The publication characteristics of FMT.

A total of 13,679 publication records met the search criteria primitively, of which 11,972 records were articles and review articles that were used finally for the analysis and visualization (Figure 1). As shown in Figure 2, these records were published between 1980 and 2022, a growing trend in publication was observed, indicating the increasing attention and interest in the FMT field. The annual publications began rapidly growing from 1991, more than 1,000 papers were published annually from 2019. Of these records, articles accounted for around 83% of document type (Figure 2), indicating a larger emphasis on original studies in the field of FMT.

### Web of Science categories

**Figure 3:**
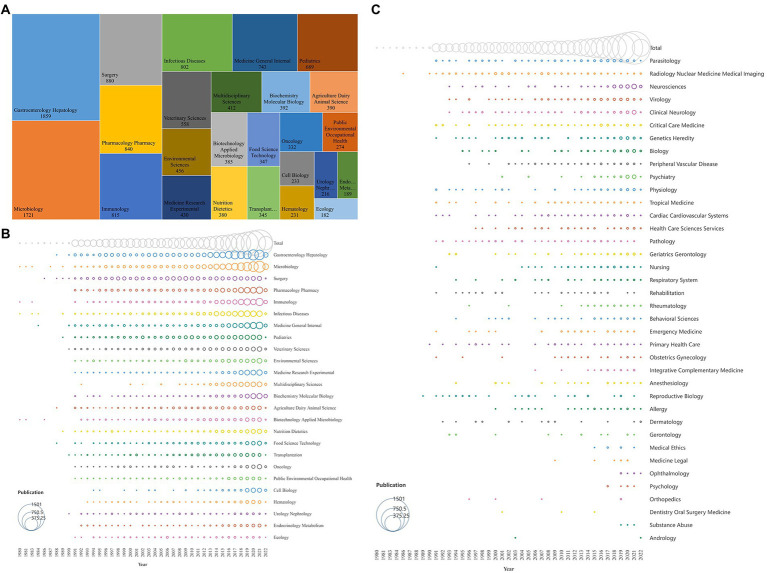
The Web of Science categories and the trend of their annual publications. **(A)** Top 25 categories of publication about FMT. **(B)** Annual publications and trend of the top 25 categories. **(C)** Annual publications and trend of other categories related to clinical medicine. The size of the circle represents the number of annual publications in each category.

All the analyzed records were divided into 173 entries of the Web of Science categories, among which gastroenterology hepatology was the largest, accounting for 15.5% of the records, followed by microbiology, surgery, pharmacology pharmacy, and immunology, etc. Top 25 categories were shown in Figure 3A, and the trend of their annual publications was shown in Figure 3B. In the remaining 148 entries, 38 were closely related to clinical medicine, and the trend of their annual publications was shown in Figure 3C. From these figures above, we could clearly find that most of the top 25 categories were the most classic and persistent research fields and also the hotspots of FMT research at the present. In addition, the number of publications in neuroscience, clinical neurology, psychiatry had increased significantly in the past three years, which may has become new research hotspots in the fields of FMT.

### Distribution and Co-authorship analysis of countries/regions

**Figure 4:**
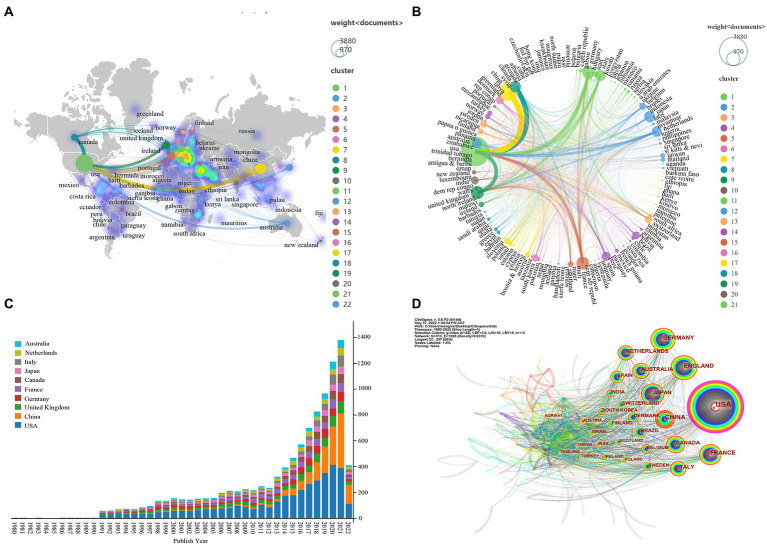
The global distribution and cooperation network of FMT research. **(A)** The global distribution of FMT research. The size of the circle represents the number of total publications in different countries, the width of the lines between different countries represents the strength of their cooperation. **(B)** The cooperation network of FMT research in different countries. The size of the circle represents the number of total publications in different countries, the width of the lines between different countries represents the strength of their cooperation. **(C)** The annual publications and trends of the top 10 countries. **(D)** The total citations of publications in different countries. The overall size of the circle represents the number of publications in different countries. Each colored circle (tree ring history) represents the number of publications published by that country in a single time slice. The width of the lines between different countries represents the strength of their cooperation; The outermost purple circle represents the country has a significant role in the FMT field.

All publications in the field of FMT were distributed among 147 countries/regions, the global distribution and cooperation of these major countries were shown, respectively, in Figures 4A,B. The production of the USA ranked the first with 3,880 documents by far, followed by the China, United Kingdom, and Germany. The top 20 countries with the most publications and their total link strength were shown in Table 1, the USA was also one of the most cooperative countries in the FMT research, and it cooperated closely with China and other countries.

**Table 1:** Characteristics of the top 20 countries with the most publications.

Num	Country	Publications	Citations	Average citations	Total link strength	Betweenness centrality
1	United States	3,880	176,555	46	1834	0.39
2	China	1,539	28,653	19	552	0.00
3	United Kingdom	794	37,777	48	969	0.07
4	Germany	770	28,102	36	843	0.04
5	France	737	34,413	47	740	0.07
6	Canada	592	26,256	44	650	0.02
7	Japan	576	13,241	23	245	0.02
8	Italy	537	18,377	34	606	0.02
9	Netherlands	492	27,099	55	647	0.03
10	Australia	445	17,727	40	438	0.02
11	Spain	391	13,181	34	450	0.02
12	Denmark	295	14,000	47	365	0.03
13	Brazil	278	5,734	21	130	0.01
14	India	272	4,713	17	178	0.01
15	Switzerland	260	10,783	41	395	0.05
16	Sweden	245	14,843	61	390	0.04
17	South Korea	240	4,819	20	136	0.01
18	Belgium	218	9,391	43	315	0.02
19	Finland	162	12,255	76	256	0.02
20	Poland	159	2,577	16	151	0.00

Total link strength, generated by VOSviewer 1.6.18 software, it indicates the strength or closeness of the country’s cooperation with other countries in the field of FMT; Betweenness centrality, generated by CiteSpace 5.8 software, it represents the influence or contribution of the country in the FMT field, and greater than 0.1 means that the country has an important contribution or a great influence.

The trends of the annual publication of the top 10 countries were shown in Figure 4C. The USA was one of the earliest countries to study FMT, and its publications increased significantly since 1991, which make it the country with the most annual publications between 1991 and 2020. As a rising star, China’s research boom on FMT mainly started after 2014, and its annual publications surpassed that of the USA in 2021. The trends of the annual publication relation to medicine of the top 10 countries were shown in Supplementary Figure 1, it was similar compared with Figure 4C.

The total citations of the USA were extremely outstanding, followed by the United Kingdom, France, and China, etc. (Figure 4D; Table 1). As shown in Figure 4D, the United States was the only country marked with purple circles and had strongest betweenness centrality (0.39), which means it played a key role in the field of FMT. Europe was not only one of the regions with the largest number of countries conducting FMT research (Figure 4A), but also had highest average citations in many countries, such as the Finland (Chen et al., 2019), Sweden (El-Salhy et al., 2020), Netherlands (Ianiro et al., 2018a), and United Kingdom (Wilson et al., 2019), etc. (Table 1). Although the number of publications in China had increased rapidly in recent years, the total citations, especially the average citations, were relatively low, and its betweenness centrality is 0. These results indicated that the quality of China research needs to be improved further.

### Distribution and Co-authorship analysis of institutions

A total of 10,019 institutions contributed to the research on FMT. The characteristics of the top 20 institutions with most publications was shown in Table 2, and ten of them located in the United States, 3 in China, 2 in Canada, and others located in the Denmark, Finland, Netherlands, France, and Brazil. The institution with the most publications (128) was the Univ Minnesota, and the institution with the highest average citations (115) was the Harvard Univ, both of which are located in the USA. The Co-authorship network of major institutions (1%) was shown in Supplementary Figure 2. The institutions marked with purple circles, including the Harvard Med Sch (0.18) and Univ Helsinki (0.1) had strongest betweenness centrality, which means they played key roles in the field of FMT.

**Table 2:** The characteristics of the top 20 institutions based on publications.

No.	Institutions	Country	Publications	Citations	Average citations	Total link strength	Betweenness centrality
1	Univ Minnesota	United States	128	9,750	76	229	0.04
2	Harvard Med Sch	United States	120	7,423	62	398	0.18
3	Univ Copenhagen	Denmark	119	7,674	64	243	0.04
4	Univ Helsinki	Finland	111	6,853	62	194	0.10
5	Mayo Clin	United States	111	8,203	74	225	0.05
6	Zhejiang Univ	China	100	2,245	22	109	0.05
7	Univ Amsterdam	Netherlands	99	7,921	80	204	0.01
8	Univ Washington	United States	97	6,767	70	211	0.01
9	Harvard Univ	United States	95	10,920	115	200	0.05
10	Univ Michigan	United States	94	5,938	63	166	0.06
11	Univ Alberta	Canada	92	4,562	50	276	0.06
12	Univ Toronto	Canada	92	3,370	37	238	0.05
13	Univ Calif Davis	United States	83	2,432	29	115	0.02
14	Baylor Coll Med	United States	82	5,627	69	171	0.00
15	Inra	France	80	6,105	76	115	0.00
16	Chinese Acad Sci	China	79	2010	25	160	0.00
17	Nanjing Med Univ	China	79	1,631	21	115	0.03
18	Massachusetts Gen Hosp	United States	76	5,188	68	212	0.01
19	Univ Calif San Francisco	United States	76	4,924	65	186	0.03
20	Univ São Paulo	Brazil	72	941	13	54	0.00

Total link strength, it indicates the strength or closeness of the institution’s cooperation with other institutions in the field of FMT; Betweenness centrality, it represents the influence or contribution of the institution in the FMT field, and greater than 0.1 means that the institution has an important contribution or a great influence.

### Distribution and Co-authorship analysis of authors

**Figure 5:**
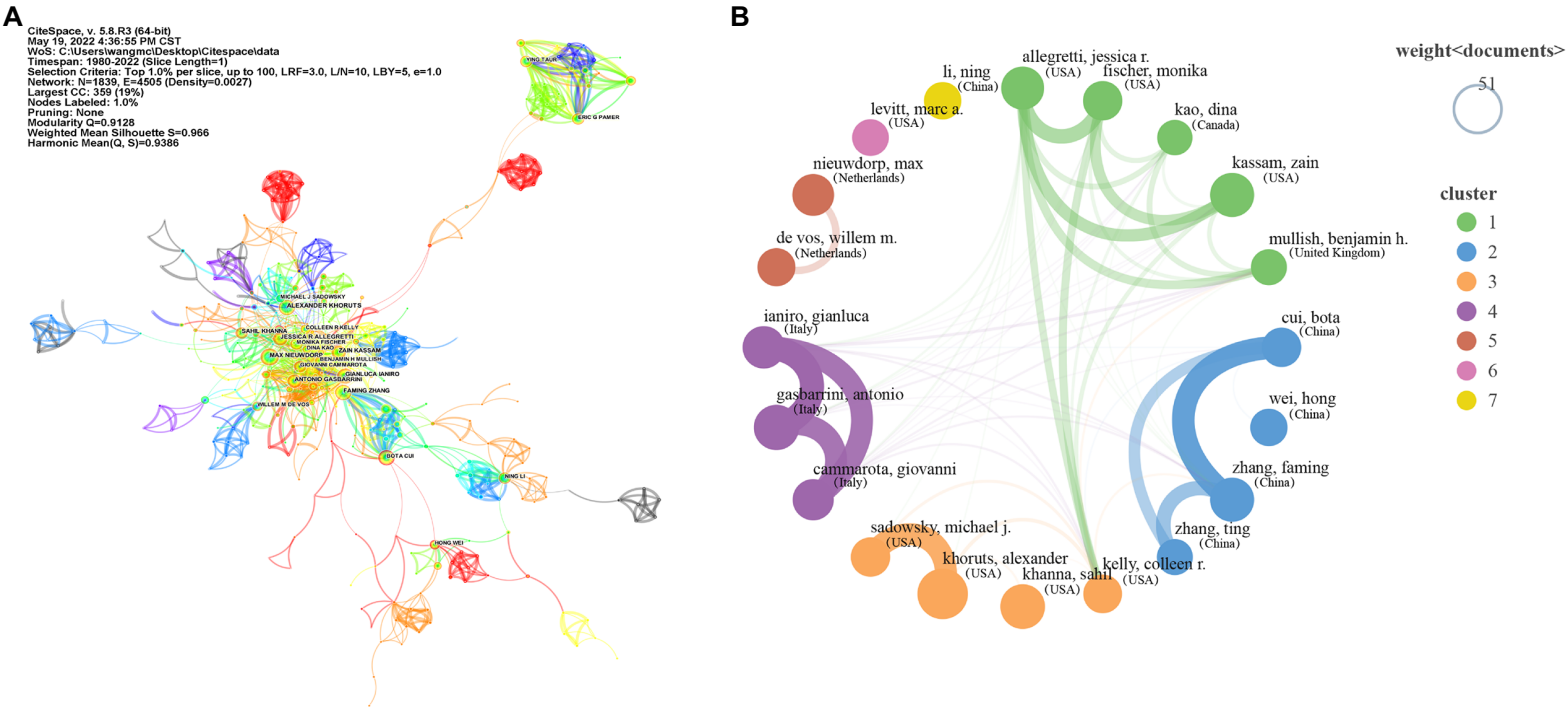
Distribution and Co-authorship analysis of authors. **(A)** The collaborations among the main authors and their teams on FMT. Each dot or circle represents an author, authors with the same color may be from the same research team; the line between them represents a collaborative relationship, and the width of the lines represents the strength of their cooperation. **(B)** The cooperative relationships of the top 20 authors with each other. The size of the circle represents the number of publications of different authors; the width of the lines between different authors represents the strength of their cooperation; authors with the same color may be from the same research team.

A total of 58,460 authors contributed to the research on FMT. The characteristics of the top 20 authors with most publications was shown in Table 3, eight of them in the USA, 5 in China, 3 in Italy, 2 in Netherlands, and others in United Kingdom and Canada. Among them, the author with highest average citation was De Vos WM (159), who worked in the Wageningen Univ of Netherlands, followed by Nieuwdorp M (116) and Sadowsky MJ (116), they worked, respectively, in the Univ Amsterdam of Netherlands and Univ Minnesota of the USA. The collaborations among the lead authors (1%) and their teams on FMT were shown in the Figure 5A. We found that most of the top 20 authors had cooperative relationships with each other (Figure 5B). The main cooperative networks of the top 20 authors with other researchers were shown, respectively, in Supplementary Figure 3.

**Table 3:** The characteristics of the top 20 authors based on publications.

No.	Author	Country	Institutions	Publications	Citations	Average citations	Total link strength
1	Khoruts, Alexander	United States	Univ Minnesota	51	4,994	98	161
2	Gasbarrini, Antonio	Italy	Univ Cattolica Sacro Cuore	41	1,389	34	148
3	Khanna, Sahil	United States	Mayo Clin	40	925	23	72
4	Kassam, Zain	United States	MIT	39	2,668	68	150
5	Zhang, Faming	China	Nanjing Med Univ	39	1,062	27	224
6	Allegretti, Jessica R.	United States	Harvard Med Sch	37	1,171	32	157
7	Ianiro, Gianluca	Italy	Univ Cattolica Sacro Cuore	36	1,298	36	149
8	Nieuwdorp, Max	Netherlands	Univ Amsterdam	35	4,064	116	76
9	Cammarota, Giovanni	Italy	Univ Cattolica Sacro Cuore	34	1,114	33	145
10	Cui, Bota	China	Nanjing Med Univ	31	826	27	186
11	Fischer, Monika	United States	Indiana Univ	31	812	26	123
12	Sadowsky, Michael J.	United States	Univ Minnesota	31	3,605	116	107
13	Kelly, Colleen R.	United States	Brown Univ	30	1967	66	97
14	De Vos, Willem M.	Netherlands	Wageningen Univ	29	4,625	159	68
15	Li, Ning	China	Nanjing Univ	28	727	26	99
16	Wei, Hong	China	Third Mil Med Univ	28	999	36	70
17	Levitt, Marc A.	United States	Cincinnati Childrens Hosp Med Ctr	27	835	31	49
18	Mullish, Benjamin H.	United Kingdom	Imperial Coll London	26	819	32	126
19	Zhang, Ting	China	Nanjing Med Univ	26	595	23	136
20	Kao, Dina	Canada	Univ Alberta	25	1,160	46	113

Total link strength, it indicates the strength or closeness of the author’s cooperation with other authors in the field of FMT.

### Active journals analysis

**Figure 6:**
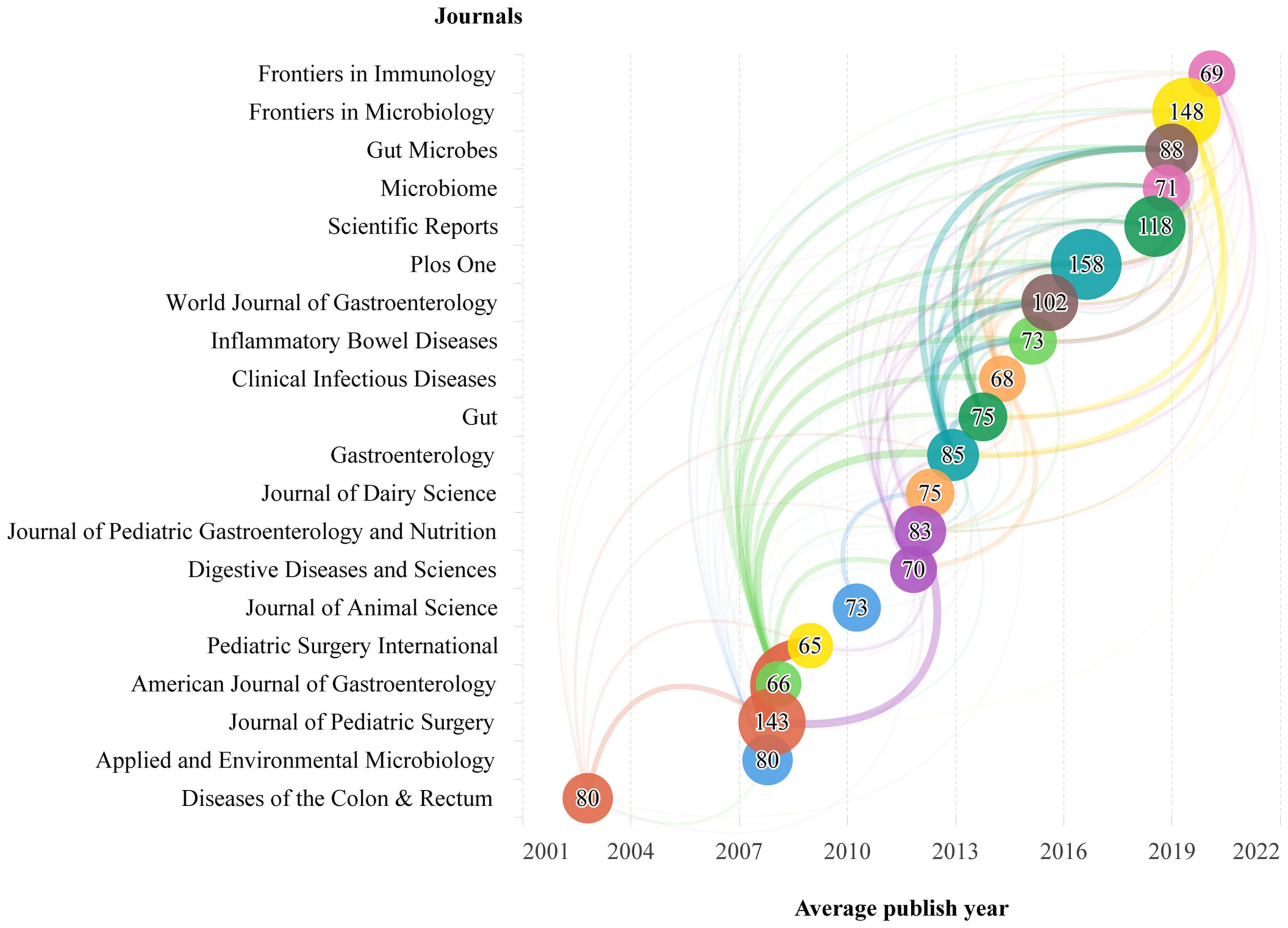
The average publish year of the top 20 journals with most publications. The size of the circle represents the total number of publications about FMT in different journals; the width of the lines between different journals represents the strength of cited each other.

A total of 2,790 journals have published documents on the subject of FMT. The characteristic of the top 20 journals with most publications was shown in Supplementary Table 2. Journal with the most publications was the Plos One (158), followed by the Frontiers in Microbiology (148), Journal of Pediatric Surgery (143), and Scientific Reports (118). Of the top 20, journal with the highest average citations was the Gastroenterology (185), followed by the American Journal of Gastroenterology (124), and Gut (104). In recent years, the following journals have begun to focus on the FMT research, including the Frontiers in Immunology, Frontiers in Microbiology, Gut Microbes, and Microbiome, etc. (Figure 6).

### Co-cited references analysis

A total of 327,028 references cited by 11,972 publications were identified by the software of VOSviewer. The top 10 most-cited references (Caporaso et al., 2010; Bakken et al., 2011; Gough et al., 2011; Vrieze et al., 2012; Kassam et al., 2013; Surawicz et al., 2013; van Nood et al., 2013; Moayyedi et al., 2015; Rossen et al., 2015; Paramsothy et al., 2017) were shown in Table 4, they were published between 2011 and 2017, and four of them were reviews. Five (Bakken et al., 2011; Gough et al., 2011; Kassam et al., 2013; Surawicz et al., 2013; van Nood et al., 2013) of the top 10 references were on the topic of FMT for the treatment of *Clostridium difficile* infection (CDI), and they were all published before 2013. Three (Moayyedi et al., 2015; Rossen et al., 2015; Paramsothy et al., 2017) of them was for ulcerative colitis (UC), one (Vrieze et al., 2012) for metabolic syndrome, and one (Caporaso et al., 2010) for QIIME, which was an analysis tool for high-throughput community sequencing data.

**Table 4:** The top 10 most-cited references.

No.	Authors	Year, journal, title	Citations	Topics	Types
1	Van Nood E	2013, N Engl J Med, Duodenal infusion of donor feces for recurrent *Clostridium difficile*	1,065	Recurrent *Clostridium difficile*	Clinical trial
2	Moayyedi P	2015, Gastroenterology, Fecal microbiota transplantation induces remission in patients with active ulcerative colitis in a randomized controlled trial	542	Ulcerative colitis	Clinical trial
3	Kassam Z	2013, Am J Gastroenterol, Fecal microbiota transplantation for *Clostridium difficile* infection: systematic review and meta-analysis	404	*Clostridium difficile* infection	Meta analysis (Review)
4	Vrieze A	2013, Gastroenterology, Transfer of intestinal microbiota from lean donors increases insulin sensitivity in individuals with metabolic syndrome	404	Metabolic syndrome	Clinical trial
5	Gough E	2011, Clin Infect Dis, Systematic review of intestinal microbiota transplantation (fecal bacteriotherapy) for recurrent *Clostridium difficile* infection	400	Recurrent *Clostridium difficile*	Review
6	Surawicz CM	2013, Am J Gastroenterol, Guidelines for diagnosis, treatment, and prevention of *Clostridium difficile* infections	396	*Clostridium difficile* infection	Review
7	Rossen NG	2015, Gastroenterology, Findings from a randomized controlled trial of fecal transplantation for patients with ulcerative colitis	395	Ulcerative colitis	Clinical trial
8	Caporaso JG	2010, Nat Methods, QIIME allows analysis of high-throughput community sequencing data	385	QIIME	Analysis method
9	Paramsothy S	2017, Lancet, Multidonor intensive faecal microbiota transplantation for active ulcerative colitis: a randomized placebo-controlled trial	391	Ulcerative colitis	Clinical trial
10	Bakken JS	2011, Clin Gastroenterol Hepatol, Treating *Clostridium difficile* infection with fecal microbiota transplantation	373	*Clostridium difficile* infection	Review

Considering that the top 10 most-cited references were published in an older time, we analyzed and summarized the top 20 most-cited references (DeFilipp et al., 2018, 2019; Gopalakrishnan et al., 2018; Halkjær et al., 2018; Ianiro et al., 2018a,b; Routy et al., 2018; Smillie et al., 2018; Suez et al., 2018; Taur et al., 2018; Wang et al., 2018; Zhang et al., 2018; Zuo et al., 2018; Allegretti et al., 2019; Bolyen et al., 2019; Costello et al., 2019; Kang et al., 2019; Paramsothy et al., 2019; Wilson et al., 2019; El-Salhy et al., 2020) published in the last 5 years, which were shown in Table 5. Most of them were clinical trial and were published between 2018 and 2020. It is remarkable that their topics were completely different from those above (Table 4). Some new topics about FMT may have become hotspots and potential trends in recent years, which included drug-resistant bacteremia (safety of FMT; DeFilipp et al., 2019), tumors (Gopalakrishnan et al., 2018; Routy et al., 2018), irritable bowel syndrome (Halkjær et al., 2018; El-Salhy et al., 2020), antibiotics-associated dysbiosis (Suez et al., 2018; Taur et al., 2018), autism (Kang et al., 2019), allogeneic hematopoietic cell transplantation (DeFilipp et al., 2018), super-donor (Wilson et al., 2019), bacterial engraftment (Smillie et al., 2018), and bacteriophage transfer (Zuo et al., 2018), etc. However, the topics that have not changed included *Clostridium difficile* infection (Ianiro et al., 2018a,b), ulcerative colitis (Costello et al., 2019; Paramsothy et al., 2019), and QIIME (Bolyen et al., 2019).

**Table 5:** The top 20 most-cited references published in the last 5  years.

No.	Authors	Year, journal, title	Citations	Topics	Types
1	Defilipp Z	2019, N Engl J Med, Drug-resistant *E. coli* bacteremia transmitted by fecal microbiota transplant	226	Drug-Resistant bacteremia	Case report
2	Costello SP	2019, JAMA, Effect of fecal microbiota transplantation on 8-Week remission in patients with ulcerative colitis: a randomized clinical trial	214	Ulcerative colitis	Clinical trial
3	Routy B	2018, Science, Gut microbiome influences efficacy of PD-1-based immunotherapy against epithelial tumors	203	Tumor	Clinical trial
4	Gopalakrishnan V	2018, Science, Gut microbiome modulates response to anti-PD-1 immunotherapy in melanoma patients	171	Tumor	Clinical trial
5	Halkjaer SI	2018, Gut, Faecal microbiota transplantation alters gut microbiota in patients with irritable bowel syndrome: results from a randomized, double-blind placebo-controlled study	98	Irritable bowel syndrome	Clinical trial
6	Wilson BC	2019, Front Cell Infect Microbiol, The super-donor phenomenon in fecal microbiota transplantation	94	Super-donor	Review
7	Suez J	2018, Cell, Post-antibiotic gut mucosal microbiome reconstitution is impaired by probiotics and improved by autologous FMT	87	Antibiotics-associated dysbiosis	Clinical trial
8	Wang YH	2019, Nat Med, Fecal microbiota transplantation for refractory immune checkpoint inhibitor-associated colitis	87	Inhibitor-associated colitis	Case report
9	Bolyen E	2019, Nat Biotechnol, Reproducible, interactive, scalable and extensible microbiome data science using QIIME 2	85	QIIME	Analysis method
10	allegretti jr	2019, Lancet, The evolution of the use of faecal microbiota transplantation and emerging therapeutic indications	77	Faecal microbiota transplantation	Review
11	Paramsothy S	2019, Gastroenterology, Specific bacteria and metabolites associated with response to fecal microbiota transplantation in patients with ulcerative colitis	76	Ulcerative colitis	Clinical trial
12	Ianiro G	2018b, Aliment Pharmacol Ther, Randomized clinical trial: faecal microbiota transplantation by colonoscopy plus vancomycin for the treatment of severe refractory *Clostridium difficile* infection-single versus multiple infusions	73	*Clostridium difficile* infection	Clinical trial
13	Ianiro G	2018a, United European Gastroenterol J, Efficacy of different faecal microbiota transplantation protocols for *Clostridium difficile* infection: a systematic review and meta-analysis	73	*Clostridium difficile* infection	Meta analysis (Review)
14	Smillie CS	2018, Cell Host Microbe, Strain tracking reveals the determinants of bacterial engraftment in the human gut following fecal microbiota transplantation	73	Bacterial Engraftment and efficacy	Clinical trial
15	Zuo T	2018,Gut, Bacteriophage transfer during faecal microbiota transplantation in *Clostridium difficile* infection is associated with treatment outcome	73	Bacteriophage transfer and efficacy	Clinical trial
16	Defilipp Z	2018, Blood Adv, Third-party fecal microbiota transplantation following allo-HCT reconstitutes microbiome diversity	70	Allogeneic hematopoietic cell transplantation	Clinical trial
17	Kang DW	2019, Sci Rep, Long-term benefit of Microbiota Transfer Therapy on autism symptoms and gut microbiota	70	Autism	Clinical trial
18	Zhang Fm	2018, Protein Cell, Microbiota transplantation: concept, methodology and strategy for its modernization	70	Faecal microbiota transplantation	Review
19	El-salhy M	2020, Gut, Efficacy of faecal microbiota transplantation for patients with irritable bowel syndrome in a randomized, double-blind, placebo-controlled study	69	Irritable bowel syndrome	Clinical trial
20	Taur Y	2018, Sci Transl Med, Reconstitution of the gut microbiota of antibiotic-treated patients by autologous fecal microbiota transplant	69	Antibiotics-associated dysbiosis	Clinical trial

**Figure 7:**
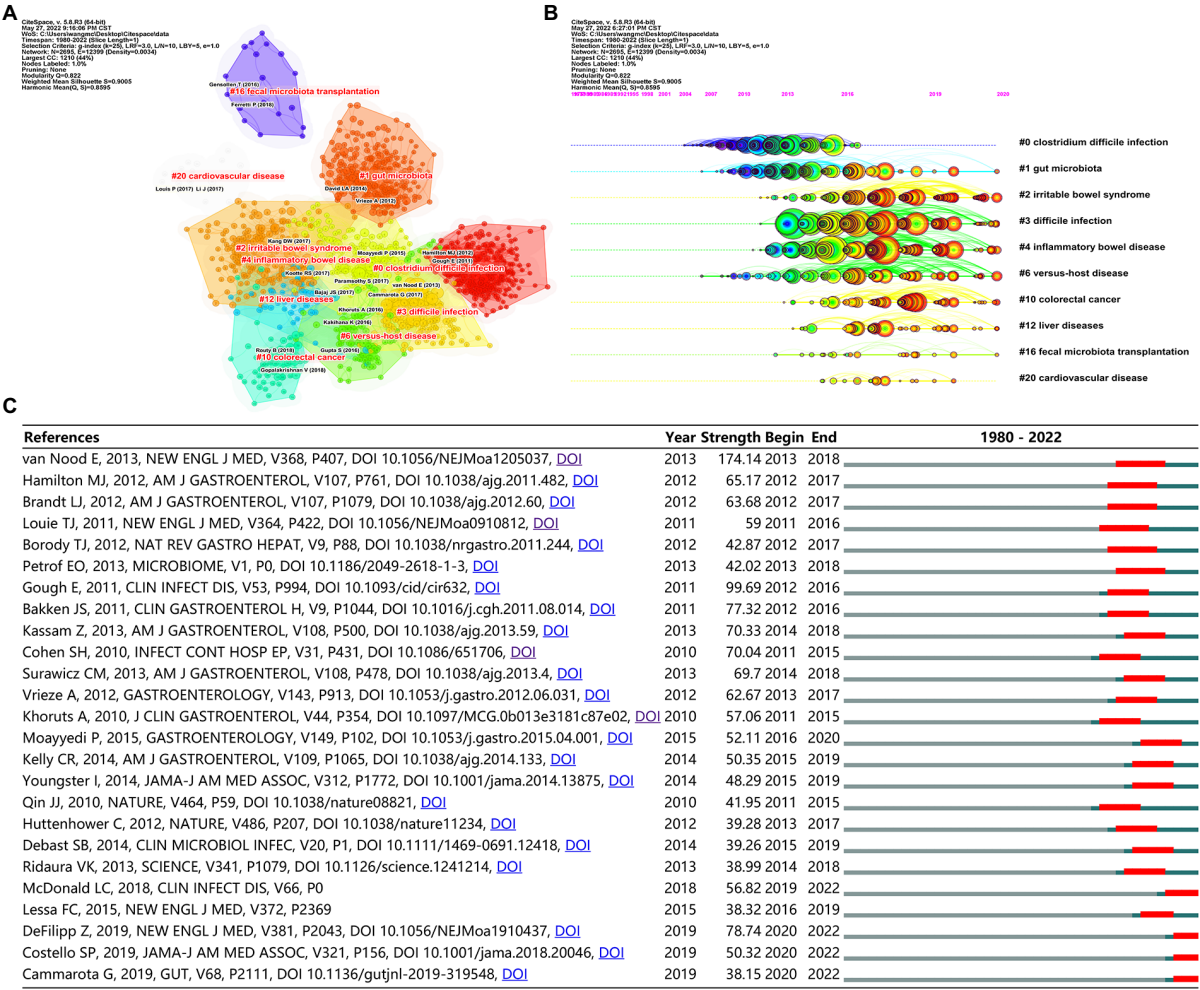
Co-cited references analysis. **(A)** The 10 major clusters of references. Each circle represents a reference, and circles with the same color represent a cluster with the same topic. **(B)** Timeline view of the 10 major clusters. Each circle represents a reference, and the circle on the same line represents a cluster with the same topic; The position of each circle represents the time when it was first cited, and the size of the circle represents the total number of it was cited. Each colored circle (tree ring history) represents the citations in a single time slice. **(C)** The top 25 references with the strongest citation bursts. The “Strength” represents the strength of citation bursts, the strength value is proportional to the bursts.

Total 10 major clusters (*Q* = 0.82, *S* = 0.90, *Q*/*S* = 0.89) were generated from the co-citation networks of references after cluster analysis by the software of CiteSpace (Figure 7A), and the cluster nomenclature may reflect the study hotspots and frontiers in FMT field. The largest cluster (2,695 Nodes, 44%) was #0 *Clostridium difficile* infection, followed by #1 gut microbiota, #2 irritable bowel syndrome, #3 difficile infection, #4 inflammatory bowel disease, #6 versus-host disease, #10 colorectal cancer, #12 liver diseases, #16 fecal microbiota transplantation, and #20 cardiovascular disease.

Timeline view of the 10 major clusters was shown in Figure 7B, which presented the cluster topics at different intervals over time. We found that most of the references in the largest cluster #0 *Clostridium difficile* infection were cited before 2016, but the references of another similar cluster #3 difficile infection were widely cited in recent years. In addition, references in these clusters, such as #2 irritable bowel syndrome, #3 difficile infection, #4 inflammatory bowel disease, #6 versus-host disease, #10 colorectal cancer, and #12 liver diseases, have also been widely cited in recent years.

The top 25 references with the strongest citation bursts were also identified *via* bursts analysis with the CiteSpace (Figure 7C), which was another method for determining research hotspots. The details of these 25 references were listed in Supplementary Table 3. Among them, 15 references were for the topics of *Clostridium difficile* infection, 2 for ulcerative colitis, 1 for metabolic syndrome, 1 for drug-resistant bacteremia, 2 for the practice guideline of fecal microbiota transplantation, and 4 for others.

### Keyword Co-occurrence analysis

There were 326 keywords with occurrence frequency greater than 5, which were extracted from the author keywords by using CiteSpace. After combining the synonyms and analogous keywords, fecal microbiota transplantation was the keyword with the most occurrence frequency. Besides, the other top 25 keywords were gut microbiota, clostridium difficile, inflammatory bowel disease, ulcerative colitis, antibiotic resistance, fecal incontinence, clostridium difficile infection, colorectal cancer, crohns disease, escherichia coli, short-chain fatty acid, irritable bowel syndrome, gut-brain axis, hepatitis virus, bile acid, stem cell transplantation, biliary atresia, graft versus host disease, liver transplantation, anorectal malformation, metabolic syndrome, quality of life, risk factor, and antegrade continence enema.

**Figure 8:**
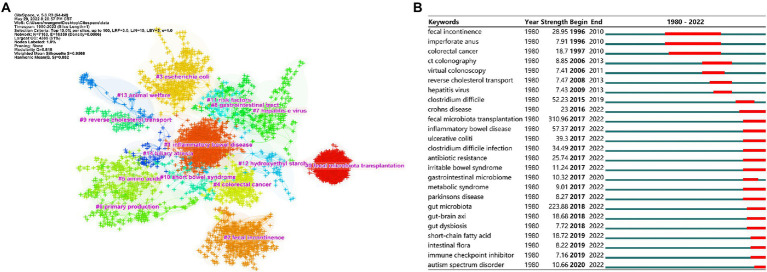
Keyword co-occurrence analysis. **(A)** The top 15 clusters of keywords. Each cross represents a keyword, and crosses with the same colors represent a cluster with the same topic. **(B)** The top 25 keywords with the strongest citation bursts. The “Strength” represents the strength of citation bursts, the strength value is proportional to the bursts. It also represents the important value of the keyword.

Total 43 clusters (*Q* = 0.81, *S* = 0.95, *Q*/*S* = 0.88) were generated after cluster analysis, the major 15 clusters were shown in Figure 8A. The largest cluster was #0 fecal microbiota transplantation (4,388 Nodes, 61%), followed by #1 inflammatory bowel disease, #2 fecal incontinence, # 3 escherichia coli, #4 colorectal cancer, #5 amino acids, #6 primary production, #7 hepatitis e virus, #8 gastrointestinal tract, # 9 reverse cholesterol transport, #10 short bowel syndrome, and #11 risk factors, etc.

The top 25 keywords with the strongest citation bursts were shown in Figure 8B. Keyword fecal microbiota transplantation had the strongest burst strength (strength = 310.96), which begun from 2017 up to now. Followed by gut microbiota (strength = 223.88, 2018–2022), inflammatory bowel disease (strength = 57.37, 2017–2022), and clostridium difficile (strength = 52.23, 2015–2019), etc. In addition, up to 2022, keywords with strongest citation bursts included ulcerative colitis (strength = 39.30, 2017–2022), clostridium difficile infection (strength = 34.49, 2017–2022), antibiotic resistance (strength = 25.57, 2017–2022), short-chain fatty acid (strength =18.72, 2019–2022), gut-brain axis (strength = 18.68, 2018–2022), and others.

### Disease keywords analysis

Keywords were extracted from all keywords by using VOSviewer software. In order to further understand the application status of FMT in different diseases, we combined keywords related to disease names and their synonyms, and then sorted them according to frequency of occurrence. Supplementary Table 4 shown the top 35 diseases for which FMT was most frequently applied. Among them, *Clostridium difficile* infection was the most common disease, followed by inflammatory bowel disease, organ transplantation, and diarrhea, ulcerative colitis, gastritis and enteritis, infectious disease, Crohn’s disease, cell transplantation, and hepatitis, etc.

### Summary of hotspots evidences

**Figure 9:**
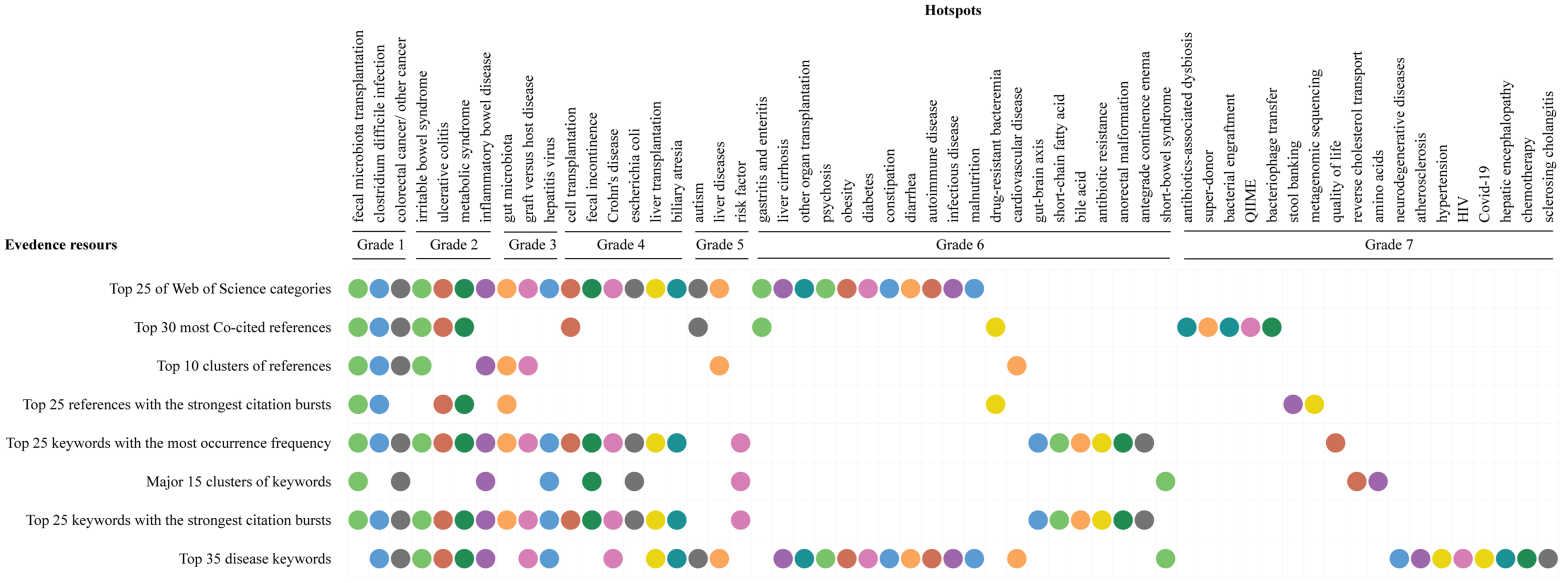
The summary of hotspots evidences.

We summarized the hotspots above and classified them into different grades according to the number of evidence sources. The evidence sources included top 25 of Web of Science categories, top 30 most Co-cited references, top 10 clusters of references, top 25 references with the strongest citation bursts, top 25 keywords with the most occurrence frequency, major 15 clusters of keywords, top 25 keywords with the strongest citation bursts, and top 35 disease keywords. The summary of hotspots evidences was shown in Figure 9, a total of 57 hotspots on FMT research were divided into 7 grades. Hotspots in grade 1 included fecal microbiota transplantation, *Clostridium difficile* infection, and colorectal cancer/other cancer. Grade 2 included irritable bowel syndrome, ulcerative colitis, metabolic syndrome, and inflammatory bowel disease. Grade 3 included gut microbiota, graft versus host disease, and hepatitis virus. Other hotspots and their grades were shown in Figure 9.

## Discussion

FMT, as a non-conventional therapy with great potential, is being applied in many clinical fields. In this study, we conducted a comprehensive bibliometric analysis of publications related to FMT, and finally gained the research hotspots and potential trends. The bibliometric analysis was performed based on publication characteristics analysis, Co-authorships analysis, Co-cited analysis, Co-occurrence analysis, and burst analysis.

After the publication characteristics analysis, we found that the researches on FMT was still in the ascendant, the number of publications was increasing year by year, and more than 1,000 papers were published annually from 2019. In this part, we analyzed the categories of all publications in the Web of Science, and regarded the top 25 categories as one of the evidence sources of hotspots on FMT (Figure 3). All publications were divided into 173 categories in the Web of Science, and most of them (51%) were in gastroenterology hepatology, microbiology, surgery, pharmacology pharmacy, and immunology.

Co-authorships analysis shown that the United States was the center of FMT research, it played a key role in the field, it was also one of the most cooperative countries with others. Although publications in China had increased rapidly in recent years, and the annual publications surpassed that of the USA in 2021, the total citations and average citations were relatively low, the quality of research needs to be improved further. Europe was another center for FMT research, with the highest average citations in many countries, such as the Finland, Sweden, Netherlands, and United Kingdom, etc. (Table 1). In addition, Frontiers in Immunology, Frontiers in Microbiology, Gut Microbes, and Microbiome were among the journals that have published many papers on the subject of FMT in recent years.

After Co-cited analysis, Co-occurrence analysis, and burst analysis, we produced another seven evidence sources and total 57 hotspots on FMT research, and these evidence sources included the top 30 most Co-cited references, top 10 clusters of references, top 25 references with the strongest citation bursts, top 25 keywords with the most occurrence frequency, major 15 clusters of keywords, top 25 keywords with the strongest citation bursts, and top 35 disease keywords. All 57 hotspots were finally divided into 7 grades according to the number of evidence sources (Figure 9).

Hotspots in grade 1 included fecal microbiota transplantation, *Clostridium difficile* infection, and colorectal cancer/other cancer, which were all given seven different evidence sources (Figure 9). Fecal microbiota transplantation (FMT) itself was still one of the hotspots, mainly due to the following reasons: (Kelly, 2013) FMT have been successfully used in a limited number of diseases, such as *Clostridium difficile* infection, and it is being eagerly attempted for the diagnosis and treatment of other diseases (Allegretti et al., 2019; Aroniadis et al., 2019; Aron-Wisnewsky et al., 2019; Kang et al., 2019; Green et al., 2020; Proença et al., 2020; Wu et al., 2022). Many factors such as characteristics of donors, types of stool material, administration routes, stool dose and frequency may affect the effectiveness and safety of FMT, but the sufficient evidences are still on the way (Borody et al., 2004; Halkjær et al., 2018; Ramai et al., 2021). The concept, methodology and strategy for its modernization are being updated and standardized (Table 5; Supplementary Table 3; Zhang et al., 2018; Cammarota et al., 2019).

*Clostridium difficile* infection is the second hotspot in grade 1, it is the most common disease for FMT applying (Supplementary Table 4). Sufficient evidences shown that FMT is highly efficacious for recurrent *Clostridium difficile* infection with response rates of around 90% (Rokkas et al., 2019; Tixier et al., 2022). In recent years, the researches of FMT on *Clostridium difficile* infection mainly focused on the following aspects: (1) Efficacy of different FMT protocols for *Clostridium difficile* infection (Table 5; Supplementary Table 3; Youngster et al., 2014; Ianiro et al., 2018a). (2) Comparison of FMT with other treatments, such as fixed bacterial mixture (Cold et al., 2022), vancomycin (Table 5; Cammarota et al., 2015; Ianiro et al., 2018b). (3) For special populations with *Clostridium difficile* infection, such as pediatric patients (Bernard et al., 2021), immunocompromised patients (Supplementary Table 3; Kelly et al., 2014), and severe or fulminant *Clostridium difficile* infection (Tixier et al., 2022). (4) The mechanisms and pharmacology of FMT for *Clostridium difficile* infection (Mullish et al., 2019; Jan et al., 2021; Khoruts et al., 2021).

The third hotspot in grade 1 was colorectal cancer/other cancer. Gut microbiota may have a close relationship with the development of colorectal cancer (Wieczorska et al., 2020), and targeted treatment of the gut microbiota could be a promising strategy for patients with colorectal cancer (Ma and Chen, 2019). In addition, mounting evidences have demonstrated that gut microbiota plays a critical role in cancer patients’ therapeutic responses to chemotherapy, radiotherapy, especially immunotherapy, including clinical efficacy and sensitivity to toxicity, and FMT is being used to modulate gut microbiota in cancer patients (Supplementary Table 3; Gopalakrishnan et al., 2018; Chen et al., 2019; Ma and Chen, 2019; Wu et al., 2019; McQuade et al., 2020).

Hotspots in grade 2 were all given six different evidence sources (Figure 9), which included inflammatory bowel disease, ulcerative colitis, irritable bowel syndrome, and metabolic syndrome. Inflammatory bowel diseases were second only to *Clostridium difficile* infection for FMT applying (Supplementary Table 4), it included ulcerative colitis and Crohn’s disease, and the Crohn’s disease was also a hotspot in the grade 4 (Figure 9). FMT is being explored as a therapeutic option for the patients with inflammatory bowel diseases and irritable bowel syndrome. The current studies mainly focus on the follow two aspects. First, many randomized controlled trials (RCTs) are being conducted in recent years, positive effects in various degrees were obtained in some RCTs, while there was no effect in the others, so the results from these RCTs are inconsistent (Zhao et al., 2020; El-Salhy et al., 2021). At the same time, almost all RCTs are small sample size studies (Aroniadis et al., 2019; Costello et al., 2019; El-Salhy et al., 2020; Zhao et al., 2020). Therefore, carrying out RCTs with large samples will be one of the research trends and hotspots in the future. Secondly, the changes of gut microbiota after FMT and the determination of disease-specific microbiota or biomarkers are of great significance for the treatment of these diseases. However, there is no consistent conclusion at present, so these will still be the hotspots and trends of future researches.

FMT has emerged as a new promising therapeutic approach in metabolic diseases, included metabolic syndrome (Vrieze et al., 2012; grade 2), obesity (Aron-Wisnewsky et al., 2019; grade 6), diabetes (Aron-Wisnewsky et al., 2019; grade 6), and cardiovascular diseases (Mehmood et al., 2021; grade 6), etc. (Supplementary Table 4). Researches of FMT in these diseases are still in the early stages, and the efficacy and mechanisms of FMT are still controversial (Aron-Wisnewsky et al., 2019). Vrieze et al. (2012) found that transfer of intestinal microbiota from lean donors increased insulin sensitivity in individuals with metabolic syndrome. Ng et al. (2022) proved that repeated FMTs enhanced the level and duration of microbiota engraftment in obese patients with T2DM, and combining lifestyle intervention with FMT led to more favorable changes in recipients’ microbiota and improvement in lipid profile and liver stiffness.

Hotspots in grade 3 were all given five different evidence sources (Figure 9), which included gut microbiota, graft versus host disease, and hepatitis virus. It is generally accepted that many diseases are characterized by gut microbiome dysbiosis (Chen et al., 2021; Yang et al., 2021), but it is difficult to identify the specific microbial patterns that could characterize different diseases. The relationship between the gut microbiome and the etiology of diseases still remains unsolved (Duvallet et al., 2017). It is also accepted that FMT could alter gut microbiota in patients with different diseases and introduce a balanced conglomerate of microorganisms. However, the relationship and the mechanisms between the gut microbiome and the effect of FMT are still unclear. Research shown that microbiota-derived metabolites, such as bile acids (grade 6), short-chain fatty acids (grade 6), and amino acids (grade 7), are proposed as possible etiological factors of some diseases, and they may provide some new avenues for the diagnosis and treatment.

Graft-versus-host disease (GvHD) is one of the life-threatening complications after allogenic hematopoietic stem cell transplant (allo-HSCT; grade 4), it is associated with up to 25% mortality (Zhang et al., 2021). Biliński et al. (2022) review shows that in the published studies to date, the overall response rate of FMT in the treatment of gastrointestinal acute GvHD could reach even 74%, with complete response accounting for 50%. At present, the clinical studies of FMT for GvHD are mainly small sample studies, the total number of patients is less than 200 (Biliński et al., 2022), and larger clinical studies are required to confirm the safety and efficacy of FMT for GvHD (Zhang et al., 2021).

FMT has therapeutic effects on various liver diseases (Gu et al., 2021b), such as viral hepatitis (grade 3), liver cirrhosis (grade 6), and other liver diseases (grade 5). In addition, there is an altered microbial composition in liver transplantation patients (grade 4) and a distinct signature of microbiota associated with the perioperative period (Lai et al., 2022), so FMT may be an intervention strategy to improve transplant outcomes.

Except for these hotspots above, others included biliary atresia, autism, psychosis, autoimmune disease, antibiotics-associated dysbiosis, gut-brain axis, drug-resistant bacteremia, HIV, Covid-19, risk factor, super-donor, and stool banking, etc. They were located in grade 5, grade 6 and grade 7 based on the number of evidence sources, but most of them have been or will become the research hotspots in the field of FMT.

This study has some limitations that need to be considered. First, the data used in this study was obtained only from the WoSCC database due to its reliability of the publications and citations. However, compared with other databases, such as PubMed and Embase, the WoSCC has fewer literatures and journals, which may increase the risk of literature selection bias. Second, the generation of hotspots is based on all types of studies. However, different types of studies do have different impacts on the field, such as RCTs, guidelines and recommendations, and the conclusions of these types of studies may be more important. Therefore, data analysis and visualization for different types of studies may be more convincing in future research. Third, research on the mechanisms of FMT is a key topic in this field, and among the 57 hotspots finally obtained, 4 are about mechanism research, which included bile acids, short-chain fatty acids, amino acids, and gut-brain axis, but we are acutely aware that these may be far from comprehensive.

In conclusion, this bibliometric analysis is expected to provide overall perspective for FMT. Based on this study, research on FMT has gained increasing attention and interest since 1991, especially in recent years. There are many hotspots about FMT, and some of them may represent the research trends in the field of FMT. These hotspots can be divided into four categories, one of which is the clinical application of FMT in various diseases. The clinical applications of FMT are comprehensive and multifaceted. Currently, *Clostridium difficile* infection is the only disease for which FMT has a clear therapeutic effect. However, there is still a lack of high-quality evidence on the efficacy and safety of FMT in other diseases, which will become a hotspot and trend of future research. The second category can be summarized as the mechanism research of FMT. Studies on the mechanism have focused on the role of gut microbiota, microbiota-derived metabolites, gut-brain axis and others, but there are no consistent conclusions at present. This will become the second hotspot and trend in future. The third category can be summarized as the standardization of FMT process, such as selection of stool donor, stool material styles, routes of FMT administration, and stool banking establishment, etc. The last category may include the pharmacology of FMT, FMT product manufacturing, etc., although they are not among the hotspots summarized in this study.

## Author contributions

MW and YZ designed the study. MW, XX, and YZ independently assessed studies for possible inclusion and collected the data. XX and SZ analyzed the data. MW and WH drafted the manuscript. YZ proofread the manuscript. All authors contributed to the article and approved the submitted version.

## Funding

This study was supported by the National Natural Science Foundation of China (82060800), Gansu Province Youth Science and Technology Fund program (20JR10RA759 and 21JR1RA149), Health Industry Science and Technology plan of Gansu Province (GSWSKY2020-30), Cuiying Scientific and Technological Innovation Program of Lanzhou University Second Hospital (CY2021-QN-A01), and Fundamental Research Funds for the Central Universities (31920200047). The funder of the study had no role in the study design, data collection, data analysis and visualization, data interpretation, or writing of the report.

## Conflict of interest

MW received research grants from the National Natural Science Foundation of China, Gansu Province Youth Science and Technology Fund program, Health Industry Science and Technology plan of Gansu Province, and Cuiying Scientific and Technological Innovation Program of Lanzhou University Second Hospital. XX received research grants from the Fundamental Research Funds for the Central Universities. WH received research grants from Gansu Province Youth Science and Technology Fund program. The remaining authors declare that the research was conducted in the absence of any commercial or financial relationships that could be construed as a potential conflict of interest.

## Publisher’s note

All claims expressed in this article are solely those of the authors and do not necessarily represent those of their affiliated organizations, or those of the publisher, the editors and the reviewers. Any product that may be evaluated in this article, or claim that may be made by its manufacturer, is not guaranteed or endorsed by the publisher.

## Supplementary material

The Supplementary material for this article can be found online at: https://www.frontiersin.org/articles/10.3389/fmicb.2022.990800/full#supplementary-material

SUPPLEMENTARY FIGURE 1 The trends of the annual publication relation to medicine of the top 10 countries. The search time is up to July 19, 2022, the number of publication relation to medicine is 9570.

SUPPLEMENTARY FIGURE 2 The Co-authorship network of major institutions. The overall size of the circle represents the number of publications in different institutions. Each colored circle (tree ring history) represents the number of publications published by that institution in a single time slice. The width of the lines between different institutions represents the strength of their cooperation; The outermost purple circle represents the institution has a significant role in the FMT field.

SUPPLEMENTARY FIGURE 3 The main cooperative networks of the top 20 authors with other researchers. The size of the circle represents the number of publications that the author has published, the line between them represents a collaborative relationship. The author's ranking is consistent with that in Table 3.

SUPPLEMENTARY TABLE 3 The top 25 references with the strongest citation bursts.
