# Supplementary material for: Global research trends and hotspots of fecal microbiota transplantation: A bibliometric and visualization study
Source: Front Microbiol. 2022 Aug 18;13:990800. doi: 10.3389/fmicb.2022.990800 (PMC9433904; doi:10.3389/fmicb.2022.990800)
Supplement: SUPPLEMENTARY FIGURE 1 — The trends of the annual publication relation to medicine of the top 10 countries. The search time is up to July 19, 2022, the number of publication relation to medicine is 9570. [file Data_Sheet_2.zip › Supplementary Table 2.docx]

Supplementary Table 2. The characteristic of the top 20 journals

| **Journals** | **Publications** | **Citations** | **Average Citations** | **IF (2020)** | **H-index** |
| --- | --- | --- | --- | --- | --- |
| Plos One | 158 | 4398 | 28 | 3.24 | 268 |
| Frontiers in Microbiology | 148 | 2835 | 19 | 5.64 | 88 |
| Journal of Pediatric Surgery | 143 | 3840 | 27 | 2.54 | 118 |
| Scientific Reports | 118 | 3794 | 32 | 4.37 | 149 |
| World Journal of Gastroenterology | 102 | 3161 | 31 | 5.74 | 129 |
| Gut Microbes | 88 | 2091 | 24 | 10.24 | - |
| Gastroenterology | 85 | 15704 | 185 | 22.68 | 368 |
| Journal of Pediatric Gastroenterology and Nutrition | 83 | 2984 | 36 | 2.83 | 121 |
| Applied and Environmental Microbiology | 80 | 4504 | 56 | 4.79 | 293 |
| Diseases of the Colon & Rectum | 80 | 4557 | 57 | 4.58 | 152 |
| Gut | 75 | 7807 | 104 | 23.05 | 262 |
| Journal of Dairy Science | 75 | 1831 | 24 | 4.03 | 166 |
| Inflammatory Bowel Diseases | 73 | 2887 | 40 | 5.32 | 128 |
| Journal of Animal Science | 73 | 1941 | 27 | 3.15 | 138 |
| Microbiome | 71 | 4032 | 57 | 14.65 | 50 |
| Digestive Diseases and Sciences | 70 | 1560 | 22 | 3.19 | 113 |
| Frontiers in Immunology | 69 | 783 | 11 | 7.56 | 84 |
| Clinical Infectious Diseases | 68 | 6638 | 98 | 9.07 | 303 |
| American Journal of Gastroenterology | 66 | 8188 | 124 | 10.86 | 234 |
| Pediatric Surgery International | 65 | 737 | 11 | 1.82 | 56 |
