# Supplementary material for: Global research trends and hotspots of fecal microbiota transplantation: A bibliometric and visualization study
Source: Front Microbiol. 2022 Aug 18;13:990800. doi: 10.3389/fmicb.2022.990800 (PMC9433904; doi:10.3389/fmicb.2022.990800)
Supplement: SUPPLEMENTARY FIGURE 1 — The trends of the annual publication relation to medicine of the top 10 countries. The search time is up to July 19, 2022, the number of publication relation to medicine is 9570. [file Data_Sheet_2.zip › Supplementary Table 4.docx]

Supplementary table 4. The top 35 diseases for which FMT was most frequently applied

| **No.** | **Disease keywords** | **Occurrences** | **Included terms** |
| --- | --- | --- | --- |
| 1 | Clostridium difficile infection | 677 | clostridioides difficile, clostridioides difficile, clostridioides difficile infection, recurrent clostridium difficile infection, c. difficile,c. difficile infection, clostridium, clostridioides difficile infection (cdi), recurrent clostridioides difficile infection, clostridium difficile colitis |
| 2 | Inflammatory bowel disease | 375 | inflammatory bowel diseases, ibd, inflammatory bowel disease (ibd) |
| 3 | Organ transplantation | 292 | transplant, solid organ transplant, solid organ transplantation, intestinal transplantation, small bowel transplantation, liver transplantation, liver transplant, organ transplantation, kidney transplantation, renal transplantation, renal transplantation, renal transplant, kidney transplant, heart transplantation |
| 4 | Diarrhea | 258 | diarrhoea, antibiotic-associated diarrhea, infectious diarrhea, chronic diarrhea, fecal incontinence |
|  | Metabolic disease | 252 | metabolic diseases, metabolic syndrome; obesity; type 2 diabetes, diabetes mellitus, type 1 diabetes, type 2 diabetes mellitus, diabetic nephropathy |
| 5 | Ulcerative colitis | 235 | ulcerative colitis (uc) |
| 6 | Gastritis and enteritis | 217 | colitis, coli, gastroenteritis, enterocolitis, enteritis, necrotizing enterocolitis, pouchitis, pseudomembranous colitis |
| 7 | Infectious disease | 186 | infections, infection control, recurrent infection, bloodstream infection, infectious disease, infectious diseases, chronic infection, bacterial infections, urinary tract infection, sepsis, bacteremia, septic shock |
| 8 | Crohn's disease | 134 | crohn disease, crohns disease |
| 9 | Cell transplantation | 134 | stem cell transplantation, allogeneic stem cell transplantation, stem cell transplant, bone marrow transplantation, hematopoietic stem cell transplantation, allogeneic hematopoietic stem cell transplantation, hematopoietic stem cell transplant, hematopoietic cell transplantation, allogeneic hematopoietic cell transplantation |
| 10 | Hepatitis | 131 | acute hepatitis, hepatitis a, hepatitis e, hepatitis e virus, hev, hepatitis e virus (hev), viral hepatitis, alcoholic hepatitis, chronic hepatitis |
| 11 | Obesity | 121 | - |
| 12 | Cancer | 114 | hepatocellular carcinoma, colorectal cancer, pancreatic cancer, cancer immunotherapy |
| 13 | Psychosis | 112 | anxiety, stress, depression, autism, autism spectrum disorder, autism spectrum disorders |
| 14 | Irritable bowel syndrome | 79 | ibs |
| 15 | Diabetes | 71 | type 2 diabetes, diabetes mellitus, type 1 diabetes, type 2 diabetes mellitus, diabetic nephropathy |
| 16 | Liver diseases | 65 | non-alcoholic fatty liver disease, nonalcoholic fatty liver disease, nonalcoholic steatohepatitis, liver diseases, chronic liver disease, alcoholic liver disease |
| 17 | Metabolic disease | 60 | metabolic diseases, metabolic syndrome |
| 18 | Biliary atresia | 60 | - |
| 19 | Graft versus host disease | 60 | gvhd, acute graft-versus-host disease, graft-versus-host disease |
| 20 | Neurodegenerative diseases | 58 | alzheimer's disease, parkinson's disease |
| 21 | Atherosclerosis | 44 | - |
| 22 | autism | 40 | autism spectrum disorder, autism spectrum disorders |
| 23 | autoimmune disease | 40 | autoimmune diseases, multiple sclerosis, rheumatoid arthritis, systemic lupus erythematosus |
| 24 | Covid-19 | 35 | - |
| 25 | Liver cirrhosis | 35 | cirrhosis |
| 26 | Constipation | 27 | slow transit constipation |
| 27 | HIV | 24 | hiv infection |
| 28 | Neuroinflammation | 23 | - |
| 29 | Cardiovascular disease | 22 | cardiovascular diseases,heart failure |
| 30 | Hepatic encephalopathy | 20 | - |
| 31 | Chemotherapy | 20 | - |
| 32 | Hypertension | 19 | - |
| 33 | Sclerosing cholangitis | 19 | primary sclerosing cholangitis |
| 34 | Short-bowel syndrome | 17 | short bowel syndrome |
| 35 | Malnutrition | 15 | - |
