# Supplementary figures and images for: Global research trends and hotspots of fecal microbiota transplantation: A bibliometric and visualization study
Source: Front Microbiol. 2022 Aug 18;13:990800. doi: 10.3389/fmicb.2022.990800 (PMC9433904; doi:10.3389/fmicb.2022.990800)

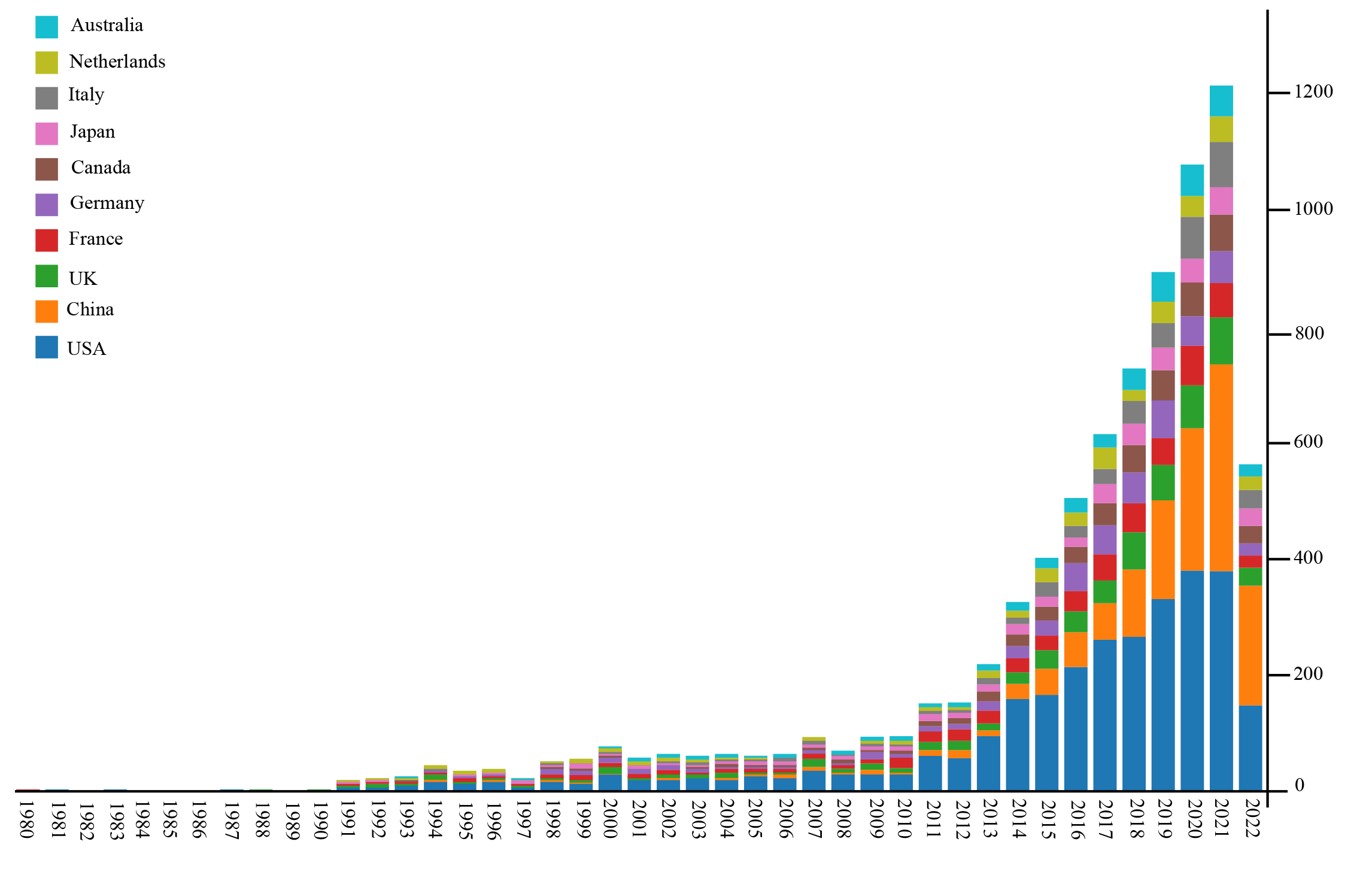

Supplement: SUPPLEMENTARY FIGURE 1 — The trends of the annual publication relation to medicine of the top 10 countries. The search time is up to July 19, 2022, the number of publication relation to medicine is 9570. [file Data_Sheet_2.zip › Supplementary Figure 1.tif]

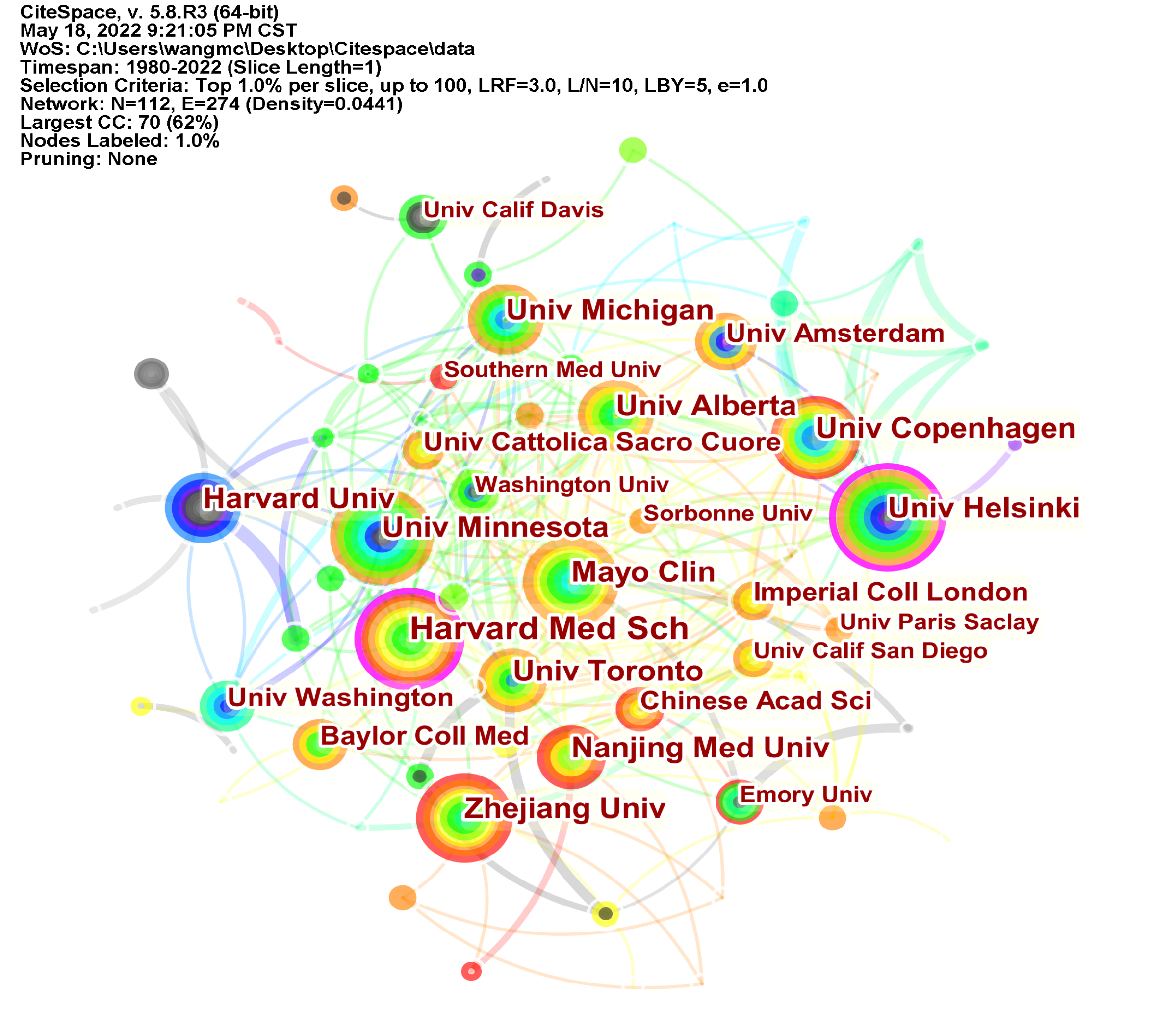

Supplement: SUPPLEMENTARY FIGURE 1 — The trends of the annual publication relation to medicine of the top 10 countries. The search time is up to July 19, 2022, the number of publication relation to medicine is 9570. [file Data_Sheet_2.zip › Supplementary Figure 2.tif]

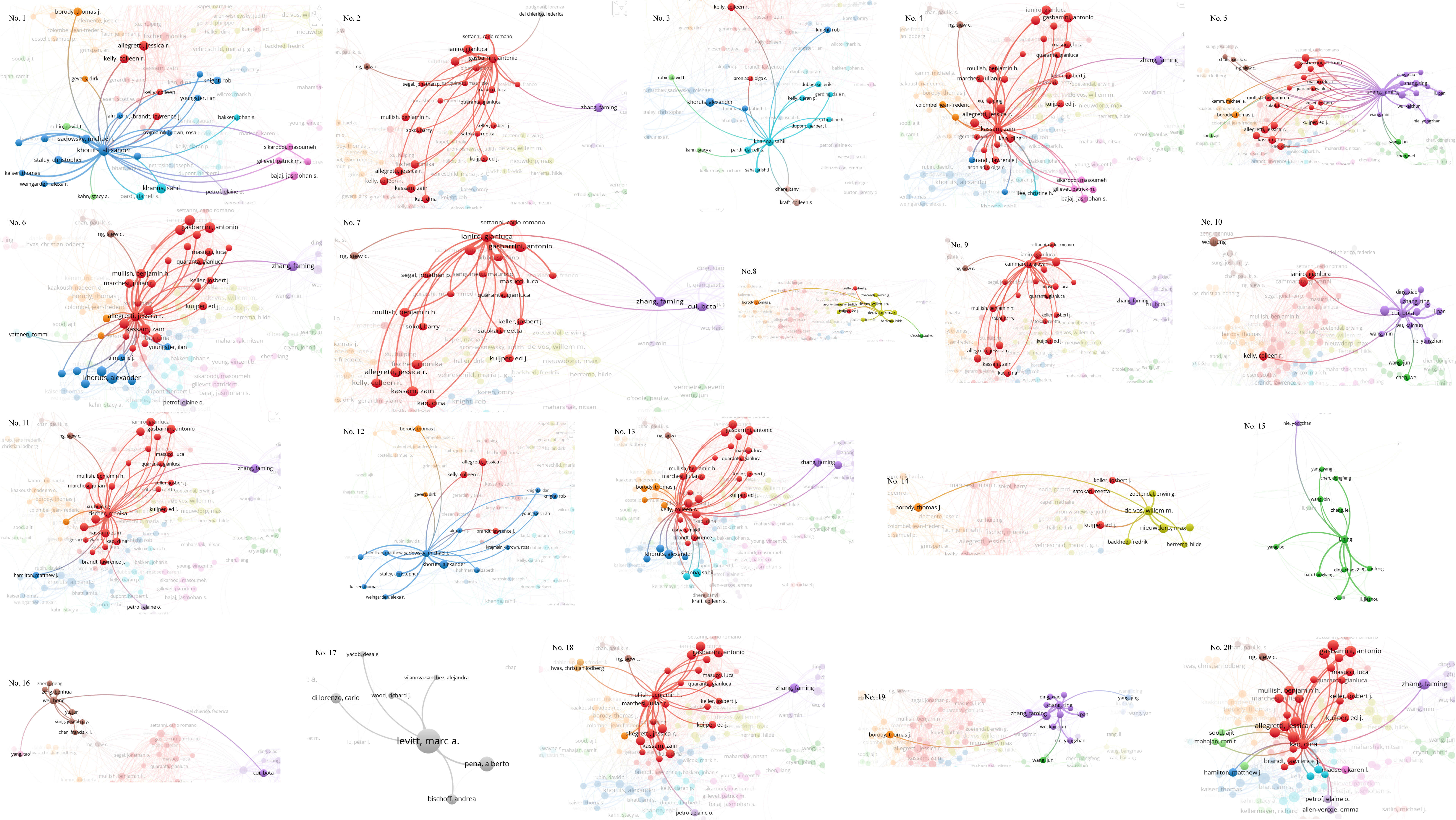

Supplement: SUPPLEMENTARY FIGURE 1 — The trends of the annual publication relation to medicine of the top 10 countries. The search time is up to July 19, 2022, the number of publication relation to medicine is 9570. [file Data_Sheet_2.zip › Supplementary Figure 3.jpg]
